# Ovarian Cancer—Epidemiology, Classification, Pathogenesis, Treatment, and Estrogen Receptors’ Molecular Backgrounds

**DOI:** 10.3390/ijms26104611

**Published:** 2025-05-12

**Authors:** Beata Smolarz, Karolina Biernacka, Honorata Łukasiewicz, Dariusz Samulak, Ewa Piekarska, Hanna Romanowicz, Marianna Makowska

**Affiliations:** 1 Laboratory of Cancer Genetics, Department of Pathology, Polish Mother’s Memorial Hospital Research Institute, Rzgowska 281/289, 93-338 Lodz, Poland; karolina.biernacka@iczmp.edu.pl (K.B.); hanna-romanowicz@wp.pl (H.R.); 2 Faculty of Medicine and Health Sciences, Department of Nursing, The President Stanisław Wojciechowski Calisia University, 62-800 Kalisz, Poland; honorata.lukasiewicz@wp.pl; 3 Department of Obstetrics and Gynecology and Gynecological Oncology, Regional Hospital in Kalisz, 62-800 Kalisz, Poland; samulakd@wp.pl; 4 Department of Obstetrics, The President Stanisław Wojciechowski Calisia University, 62-800 Kalisz, Poland; 5 Regional Hospital in Kalisz, 62-800 Kalisz, Poland; m.smol@wp.pl; 6 Department of Anesthesiology and Operative Intensive Care Medicine, Charité-Universitätsmedizin Berlin, Corporate Member of Freie Universität Berlin, Humboldt-Universität zu Berlin, 10117 Berlin, Germany; marianna.makowska@yahoo.com

**Keywords:** ovarian cancer, BRCA1/2, polymorphism, estrogens, ESR1

## Abstract

Global epidemiological reports indicate a steady increase in the tendency to develop ovarian cancer. The symptoms of ovarian cancer are non-specific, and there is no effective screening tool. Most often, surgery, chemotherapy, and radiotherapy, alone or in combination, are used to treat ovarian cancer. We have a better understanding of the biology of ovarian cancer, the genetic basis of hereditary ovarian cancer, the stage of the disease, and the role of cytoreductive surgery and more effective chemotherapy, which translates into an increase in the percentage of patients who survive 5 years after diagnosis. A growing body of evidence points to the role of genetic factors in the development of cancer. It is known that mutations in the *BRCA1* gene are responsible for an increased risk of developing ovarian cancer. The role of other genetic disorders, such as polymorphic variants, in increasing the risk of developing cancer is still being investigated. Ovarian cancer is a hormone-dependent cancer and its steroid hormones are estrogens. Estrogens affect cells through the estrogen receptors ERα and ERβ. An imbalance between ERα and ERβ receptor expression may, therefore, be a key step in estrogen-dependent carcinogenesis. In 60% of cancer cases, significantly elevated levels of ERα receptors are detected. The ERα receptor is encoded by the *ESR1* gene, so its polymorphisms can be considered molecular markers of ovarian cancer. This article discusses the epidemiology, pathogenesis, risk factors, genetic testing, treatment, and diagnosis of ovarian cancer, as well as providing an overview of standard treatment approaches and new, targeted biologic therapies.

## 1. Epidemiology of Ovarian Cancer

The number of cancer patients remains high, and it is the second leading cause of death after cardiovascular diseases [1,2,3]. Worldwide, the percentage of people developing cancer is significantly increasing.

**Figure 1:**
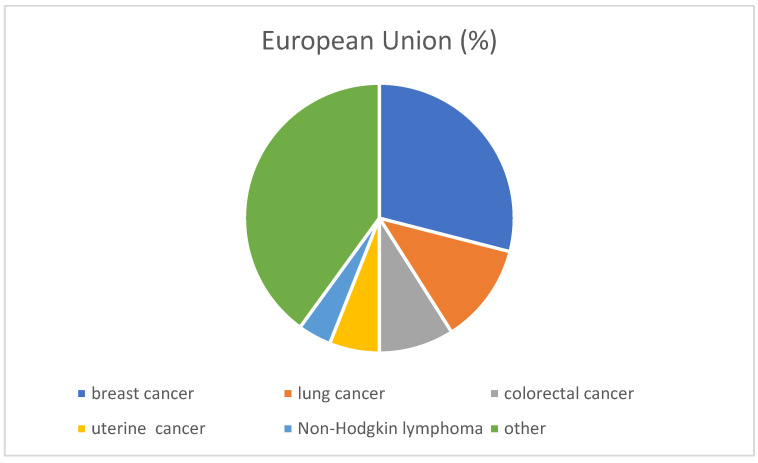
Cancer incidence in the European Union in 2020 [3].

Lung cancer is the leading cause of death caused by cancer, followed by colorectal cancer. Risk factors that increase the incidence of cancer, such as being overweight, obesity, alcohol consumption, or air pollution, are at a high level in the European Union countries. These risk factors contribute to almost half of all deaths (OECD 2021) [4]. Better prevention strategies are therefore needed [4]. Figure 1 shows the incidence of cancer in the European Union in 2020.

Around 240,000 cases of ovarian cancer are diagnosed annually in the world. It is most often diagnosed in Europe, North America, Australia, and New Zealand [5,6].

In 2022, the highest prevalence in women was recorded for breast cancer (239.4 thousand), endometrial cancer (73.5 thousand), colorectal cancer (64 thousand), thyroid cancer (47.8 thousand), cervical cancer (29.1 thousand), ovarian cancer (28.3 thousand), lung cancer (23.9 thousand), and skin melanoma (22.7 thousand) [2].

Ovarian cancer is the second-most common cancer of the female reproductive organs, right after endometrial cancer. Most ovarian cancer cases occur after menopause. Ovarian cancer mainly affects women aged 55–70. The peak of incidence occurs between the ages of 55 and 59 [7]. About 70% of ovarian cancers are detected at an advanced stage (FIGO III and IV) (The International Federation of Gynecology and Obstetrics) [8].

## 2. Risk Factors for Ovarian Cancer

**Figure 2:**
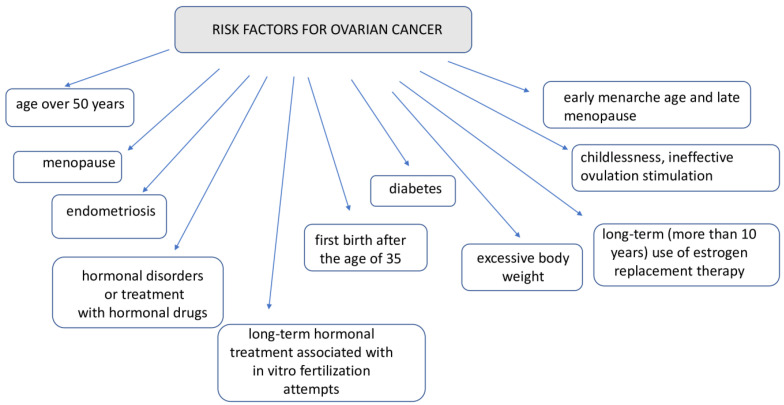
Risk factors for ovarian cancer.

Being a carrier of mutations in the BRCA1 and BRCA 2 genes is one of the most well-known risk factors for ovarian cancer; 10–15% of all ovarian cancers have a genetic basis associated with these mutations. The risk of developing ovarian cancer for BRCA1 mutation carriers is about 44%, and for BRCA2 mutation carriers, it is about 17% [2,9]. Lynch syndrome (a family history of non-polyposis colorectal cancer, a genetic disease characterized by an increased tendency to develop various cancers such as ovarian cancer, endometrial cancer, and urinary tract cancer) is responsible for about 10% of ovarian cancers [10]. Other risk factors for ovarian cancer are shown in Figure 2.

The risk of developing ovarian cancer can be reduced as a result of hysterectomy, breastfeeding, the closure of the fallopian tubes, or the use of hormonal contraception [2,11,12].

The data show a decrease in the incidence of up to 60% in women who use oral contraceptives for 10 years. Taking oral contraception over a shorter, five-year period also has a beneficial effect, reducing the incidence of disease, but only by 20–30% [13].

It has been proven that four births reduce the risk of developing the disease by about 40% [14]. Two theories explain the reduction in incidence as a result of pregnancies and childbirths, as well as the use of contraception. The first says that the beneficial effect of pregnancies and childbirth is based on the inhibition of ovulation, the consequence of which is the prevention of damage to the epithelium on the surface of the ovary. As a result of this process, there is no development of inclusion cysts, and thus the start of carcinogenesis. The second theory explains this situation by apoptosis (a natural process of programmed cell death) induced by progesterone or progestogens. As a result, cancer cells are removed from the ovary [15].

There are reports that indicate an almost twenty-fold increase in the risk of developing the disease in women undergoing the in vitro procedure, in whom the cause of infertility is unclear [16].

The aspect of diet is important. A diet rich in vegetables and fruits, especially tomatoes, reduces the likelihood of ovarian cancer by 70% [17,18].

Some histological types of ovarian cancers can develop on the basis of endometriosis. In patients with endometriosis, the risk is 1.8% [19,20]. Endometriosis is thought to affect about 11% of women of childbearing age, including 50–60% of women and adolescents suffering from pelvic pain and up to 50% of women suffering from infertility [21].

Although pelvic pain and infertility are the most well-known comorbidities of endometriosis, it is also believed that ovarian, breast, and endometrium cancer may also be associated with endometriosis. A 2021 systematic review and meta-analysis found that women with endometriosis had almost twice the risk of ovarian cancer compared to women without endometriosis, although the associations varied according to the ovarian cancer histotype [22]. There was strong evidence to support links between endometriosis and low-grade clear cell, endometrioid, and serous ovarian cancer. However, such associations have not been consistently detected in high-grade serous or mucinous neoplasms [22].

A better understanding of the relationships between the endometriosis subtypes and the ovarian cancer histotypes may provide new insights into the etiological pathways of both diseases and influence clinical decision-making regarding people with endometriosis.

## 3. Ovarian Cancer Prevention

At present, no systemic population studies are being conducted for the early detection of ovarian cancer. In the case of patients with a hereditary burden of breast cancer and ovarian cancer (who are carriers of the *BRCA1/2* gene mutation), follow-up medical and imaging examinations are recommended. As part of the first stage of care for families with a high, hereditary risk of developing breast cancer or ovarian cancer, people who have a high, hereditary risk of developing breast and ovarian cancer are identified. A detailed family history is conducted, and, if there are medical indications, genetic testing is performed. As part of the second stage, a woman with a high, hereditary risk of ovarian cancer is provided with specialist care.

The supervision of such women consists of systematic diagnostic tests and medical consultations in order to quickly detect any abnormalities. Every woman should have regular gynecological examinations. An annual gynecological examination is recommended for women over 18 years of age, and an annual rectal examination for women over 35. In this way, you can feel the ovaries and surrounding organs, checking them for shape and size.

The action recommended in scientific papers is the removal of the ovaries and fallopian tubes in *BRCA1/2* mutation carriers after procreation [23,24]. The prophylactic resection of the ovaries and fallopian tubes is most beneficial in the context of hereditary breast and ovarian cancer syndrome. This can reduce the risk of ovarian cancer by 70–85%. Preventive resection also reduces the risk of breast cancer by 54% and overall mortality by 60–70% [25,26].

The timing of ovariectomy varies depending on the mutation. This is because the average age at the time of the diagnosis of the cancer caused by *a BRCA1* gene mutation is about 10 years younger than in the case of a *BRCA2* gene mutation, and it is even later that the diagnosis is established in the case of mutations in the genes responsible for hereditary ovarian cancer (*RAD51C*, *RAD51D*, *BRIP1*). Fallopian tube resection is a solution for women who want to undergo sterilization or for women who do not agree to ovariectomy. In this case, the patient should be informed that after some time the ovaries will also have to be removed. At the moment, there is much less data on the effectiveness of tubal resection alone in this situation.

The main activities of ovarian cancer prevention include leading a healthy lifestyle and regular gynecological visits (including transvaginal ultrasound). Unfortunately, to date, it has not been possible to develop an effective screening program to detect stage I cancer, either based on imaging methods or methods using biomarkers (Ca 125, HE4, Beta2-microglobulin, transferrin, apolipoprotein, and prealbumin) [27].

## 4. Symptoms of Ovarian Cancer

Unfortunately, the symptoms of ovarian cancer are uncharacteristic and occur only at an advanced stage of the disease [28]. The symptoms usually only become apparent when the lesion is a few cm long. Symptoms of cancer include vaginal bleeding; pain in the lower abdomen; constant pain in the abdomen or pelvis; back pain in the lumbar region; problems with urination (among others due to the pressure exerted by the tumor on adjacent organs); symptoms of the digestive system, for example, bloating, enlargement of the abdominal circumference, feeling of fullness, belching, constipation, or diarrhea; a feeling of constant severe fatigue; and unexplained weight loss.

In the differential diagnosis in the above situations, the possibility of ovarian cancer should always be taken into account. The patient should be referred to a gynecologist immediately, even if a visit took place within the last 6 months. This reduces the delay in diagnosing and treating ovarian cancer.

## 5. Ovarian Cancer Diagnosis

Ovarian tumors are diagnosed on the basis of medical history and medical examinations, such as

Basic blood and urine tests.

Gynecological examination in combination with abdominal ultrasound and transvaginal ultrasound (USG-TV–transvaginal).

Examination with a genital tract speculum.

Tumor marker testing: CA125, HE4, test ROMA (Risk of OvarianMalignancyAlgorithm)—an algorithm to assess the likelihood of the ovarian cancer risk. The algorithm takes into account three elements: the risk of ovarian cancer associated with the pre- or postmenopausal period and the levels of tumor markers CA125 and HE4 [29,30]. Table 1 shows the interpretation of the ROMA test results.

Testing the level of human chorionic gonadotropin (beta HCG), alpha-fetoprotein (AFP), lactate dehydrogenase (LDH), and inhibin—determined in non-epithelial ovarian cancers.

Chest X-ray or CT scan.

Computed tomography (CT) of the abdominal cavity and pelvis.

The final confirmation of the diagnosis is based on the results of a histopathological examination. For the histopathological examination, a specimen of the neoplastic tissue is taken (laparoscopically or with an abdominal opening). In rare cases, cancer can be diagnosed by the presence of cancer cells in the fluid from the pleural cavity, peritoneum, and lymph nodes. As a result of the tests performed, it is possible to determine the stage of ovarian cancer.

## 6. Morphological Types of Ovarian Cancer

We distinguish the morphological types of ovarian cancers; cancers that arise from the epithelium of the ovary account for 90% of all ovarian cancers. Epithelial cancers, i.e., ovarian cancers, can be divided into type I and type II tumors. While type I tumors (endometrioid, clear cell, and low-grade serous and mucinous carcinomas) are less aggressive, type II tumors (mainly low-maturity and high-grade serous carcinomas) behave much more aggressively, leading to early and generalized spread. Low-grade serous carcinoma is the most common type of epithelial carcinoma, accounting for about 60–80% of ovarian cancer cases [31].

Mucinous carcinomas (5–10%), endometrial carcinomas (7–10%), clear cell carcinomas (8–12%), carcinosarcomas (5–10%) and other rarer histological types are less common. The remaining 10% are non-epithelial neoplasms [32]. Non-epithelial neoplasms are diagnosed at a low stage and most often affect women at a young age.

Non-epithelial ovarian cancers arise from germ cells (namely germline cancers; for example, ovarian germinoma, teratoma, and chorionic germ cell carcinoma) or from genital cords and their cells (gonadal tumors, granulosa tumors, and capsule cell tumors). Germcell tumors (GCTs) are non-epithelial ovarian cancers that arise from reproductive cells (those involved in the production of an egg). They appear mainly in young women and girls. The risk factors for the development of this type of cancer are

- Gonadal dysgenesis, with the 46 XY karyotype and sex-determining region Y (SRY) gene encoding a protein that determines testicular development.
- Androgen insensitivity syndrome.

Ovarian germinoma is a malignant germ cancer that develops from primary germ cells. It is one of the most common germ cell tumors of the ovary. It is a cancer with a good prognosis. It usually occurs unilaterally, although there are cases (8–15%) of bilateral occurrence. Ovarian germinoma grows rapidly and infiltrates adjacent organs. It also gives distant metastases (mainly to the lymph nodes and the lungs) and tends to recur. It most often affects women in the second or third decade of life. It is usually a tumor with a clean weave, and mixed forms are rare. Importantly, about 15–20% of cases of this cancer are detected during pregnancy or shortly after childbirth.

Ovarian germinal germblastoma is not the same disease (despite the similarity of the name of the disease) as ovarian germinoma. The former is a rare but benign ovarian tumor; the latter is malignant. Germinoma, like other ovarian cancers, is a disease that usually does not show any symptoms for a long time, and if they appear, they are often attributed to other disease entities. The main symptoms of germinoma include vaginal discharge, pain during bowel movements, lower abdominal pain, more frequent urination than usual, irregular menstruation, and a feeling of fullness in the abdomen. Some affected women observe an enlargement of the abdominal circumference, which is related to the appearance of fluid in the abdominal cavity. Some of them complain of bloating, nausea, and vomiting. Since germinoma often does not give specific symptoms, it is often detected during a routine health check-up carried out in a gynecologist’s office. After a physical examination and transvaginal ultrasound, the specialist decides on further treatment. If ovarian cancer is suspected, the patient is referred for further diagnostic tests (the determination of tumor markers, computed tomography, and magnetic resonance imaging) to determine the nature of the lesion. The treatment of ovarian germinoma first involves the surgical removal of the ovarian germinoma (together with the ovary and fallopian tube). The adjuvant treatment is usually chemotherapy or, less often, radiotherapy.

## 7. Histopathological Classification of Ovarian Cancer

About 95% of ovarian cancers originate from the epithelium covering this organ. It is a molecularly diverse group of cancers. Molecularly, they are divided into two types [33].

Type I is low-grade ovarian cancer. It accounts for 25% of all cancers. These include serous and endometrioid carcinomas, mucinous carcinomas, and clear cell carcinomas. The lesions from which these cancers develop are called borderline tumors. In type I ovarian cancers, *BRAF* and *KRAS* mutations are found in 65% of cases; additionally, mutations of other genes, such as *HER2*, *PIK3CA*, and *PTEN*, may be present. Type I ovarian cancers are characterized by slow growth and good prognoses. Five-year survival is observed in about 55% of patients [34].

Type II is a low-maturity and high-grade ovarian cancer. It is characterized by rapid growth dynamics and an aggressive course. It accounts for 75% of all cancers of this organ, which most likely originate from the distal or hyphal section of the fallopian tube. In this type of cancer, mutations in the *p53* gene (about 90%) and in the *BRCA* gene are found. Type II ovarian cancers are usually detected late, in an advanced stage of the disease, with the presence of metastases. The 5-year survival rate does not exceed 30%.

Ovarian cancers can be classified according to their molecular classification and anatomical stage. Given the heterogeneity of individual tumors, not every patient can be precisely categorized. It is suggested to use the concept of five main pathogenetically independent and histologically and molecularly different groups of ovarian cancers.

(1) High-grade serous carcinomas;
(2) Endometrioid carcinomas;
(3) Clear cell carcinomas, which often arise in the ovary and are often associated with endometriosis;
(4) Mucinous carcinomas;
(5) Low-grade serous carcinomas [35].

**Figure 3:**
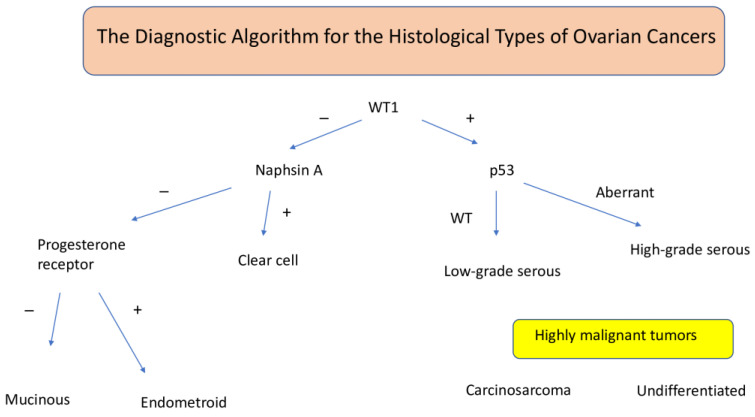
The diagnostic algorithm of the histologic types, based on additional immunohistochemistry examinations. − indicates negative; +, positive; WT, wild type.

An algorithm using molecular markers for the aforementioned five types is provided in Figure 3.

The stages of ovarian cancer have been determined according to the FIGO scale (International Federation of Gynecology and Obstetrics) in stages I–IV, where I is the least advanced disease and IV is the most advanced disease [36].

The classification of ovarian cancers in women was updated in early 2014 and for the first time also includes fallopian tube and peritoneal cancers [37]. Table 2 shows the stages of cancer, according to FIGO 2014.

Grading refers to the degree of histopathological differentiation, i.e., the assessment of the histological malignancy of the cancer, i.e., whether the cancer is metastatic or less susceptible to spreading. The assessment is based on the diversity of the structure and the cytological features of the cancer cells.

There are three types of histopathological differentiation:

- G1—well-differentiated cancer (undifferentiated cells < 5%);
- G2—moderately differentiated cancer (50% of undifferentiated cells);
- G3—undifferentiated cancer (undifferentiated cells > 50%).

## 8. Ovarian Cancer Treatment

The standard of care in ovarian cancer is surgical treatment. The qualification for the procedure is an extremely important process. Correct classification should be based on the results of imaging tests (computed tomography of the chest, abdominal cavity, and pelvis) and the experience of the operator. Performing optimal cytoreductive surgery is a fundamental step, regardless of the stage of clinical advancement, which determines the patient’s treatment plan. The primary surgery involves the complete removal of the uterus and its appendages (ovaries and fallopian tubes) and the excision of the pelvic and paraaortic lymph nodes up to the level of the left renal vein. Ovarian cancer requires combination therapy with chemotherapy with paclitaxel (a cytostatic drug) and platinum derivatives (other cytostatic drugs) [38]. The decision to qualify the patient for surgery or cytostatic treatment is made by an interdisciplinary team. As a rule, six courses of chemotherapy are administered. An exception may be made for patients with stage IA or IB cancer according to the FIGO classification, with limited grades G1 and G2. Radiotherapy is currently of limited importance in the fight against ovarian cancer. It is usually used as a palliative or symptomatic treatment [39,40]. In the case of young, childless women who want to maintain the ability to procreate, sparing treatment is used. If the disease is at an early stage, the procedure involves the unilateral removal of the diseased ovary and the fallopian tube, along with the adequate examination of the stage using surgical and microscopic methods. The survival of these patients compared to those who undergo radical surgery is favorable [41]. In a certain group of patients with early stage and well-differentiated cancer, chemotherapy may not be used after surgery. In the case of patients whose cancer is significantly advanced, yet it is not possible to remove it, chemotherapy is often initially used. When the disease is found to have withdrawn, surgery is then performed.

In the case of patients whose cancer cells are sensitive to hormones (they have hormone receptors), hormone therapy (HTH) is used. It is part of supportive treatment.

Hormone therapy in supportive treatment is aimed at slowing down the development of cancer and improving the quality of life. Hormone-sensitive cancer cells have hormone receptors. The drugs used in hormone therapy modulate signal transduction by interacting with sex hormone receptors and reduce the hormonal activity of a given tissue. Table 3 shows the options for hormonal treatment.

Hormonal drugs that have been used in the treatment of ovarian cancer include selective estrogen receptor modulators (tamoxifen), aromatase inhibitors (letrozole, analogues), and gonadotropin-releasing hormones (goserelin) [42] (Table 4).

Hormone therapy is aimed at slowing down the development of cancer and improving the quality of life. Unfortunately, hormonal treatment is associated with side effects: headaches, mood changes, weight gain, and fatigue. Rarely, serious complications, such as pulmonary embolism, arterial thrombosis, or ischemic stroke, may occur.

The maintenance treatment of ovarian cancer involves the use of PARP inhibitors. Their action focuses on blocking the repair mechanisms of cancer cells damaged during chemotherapy [43]. As a result of the accumulation of damage and the lack of regeneration mechanisms, cancer cell death occurs.

The patients disqualified from surgical treatment are the patients whose degree of fitness makes it impossible to perform surgery. They are most often characterized by pleural effusion and the histologically confirmed presence of cancer cells in the fluid. Patients who have progressed during neoadjuvant treatment are not eligible for surgical treatment. They receive chemotherapy and antiangiogenic treatment [44].

**Figure 4:**
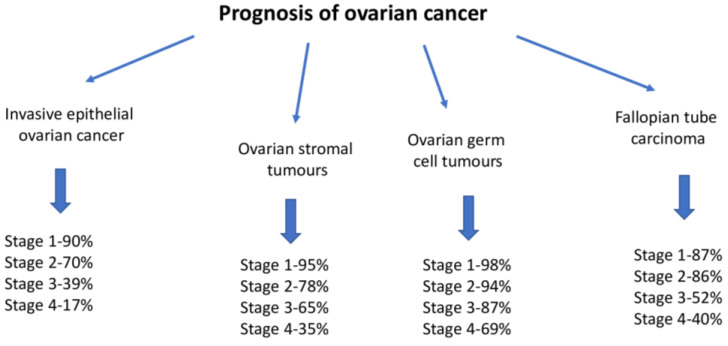
Prognoses of ovarian cancers [45].

The prognosis in patients with ovarian cancer depends on the following factors:

- The clinical stage (Figure 4).
- Whether a complete cytoreduction procedure is possible.
- Sensitivity to platinum derivatives in systemic therapy.

The individualization of treatment methods and new drug regimens improves the prognosis of ovarian cancer.

## 9. Genetic Factors

The majority of ovarian cancers (75–90%) are sporadic and develop as a result of the accumulation of somatic mutations, i.e., non-hereditary changes that are acquired successively during individual life [46].

**Figure 5:**
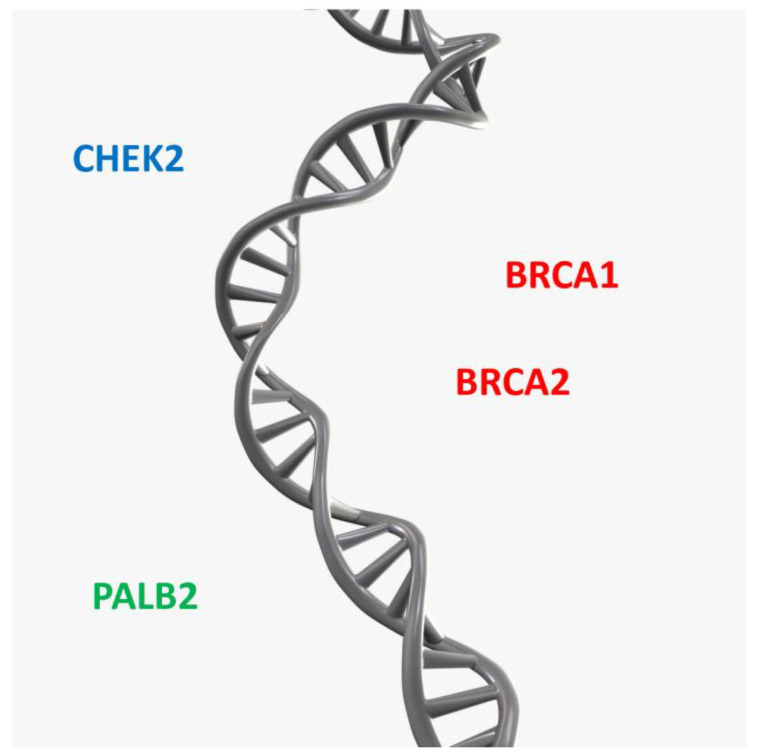
Genes associated with ovarian and breast cancer risks.

The predisposition to breast and ovarian cancer is caused by high-penetrance genes, such as *BRCA1* and *BRCA2*, which are associated with a high risk of developing the disease. Low-penetrance genes, such as *CHEK2* and *PALB2*, are also involved in the development of these diseases and are associated with a lower risk of disease (Figure 5).

These mutations are limited to the genome of cancer cells, i.e., tDNA–DNA isolated from tumor cells. Sporadic cancers are usually diagnosed in adulthood and are not related to the family history. In the case of hereditary ovarian cancers, they are associated with the carrier of mutations in the terminal *BRCA1/BRCA2* genes. These genes are responsible for the syndrome of hereditary predisposition to breast and ovarian cancer. *BRCA1* and *BRCA2* are tumor suppressor genes located on chromosomes 17q21 and 13q12, respectively, which are responsible for an increased risk of developing breast and ovarian cancer.

**Figure 6:**
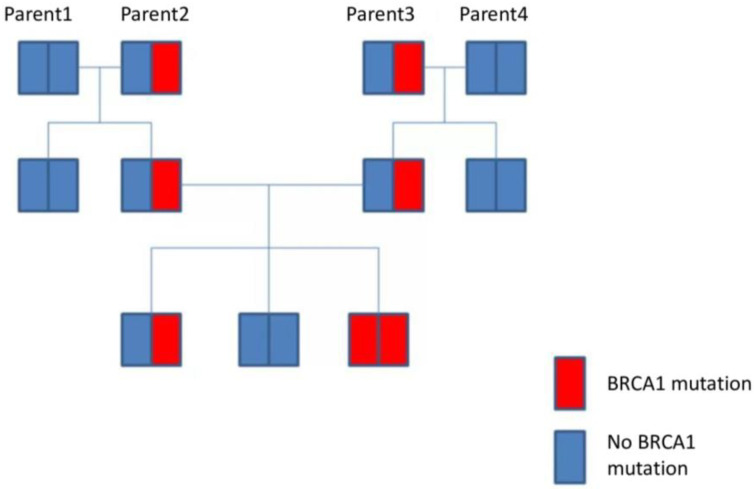
An example of a familial inheritance pattern for BRCA1 mutations.

An example of the familial inheritance of BRCA1 mutations is shown in Figure 6.

Proteins encoded by *BRCA1/2* genes are responsible for regulating cellular processes and are involved in DNA repair. One type of DNA strand repair is homologous recombination repair (HRR). Repair by homologous recombination involves the replacement of similar or identical parts of chromosomes. Errors and damage accumulate in the absence of repair of the genetic material. The result of the accumulation of errors is the transformation of a healthy cell into a cancer cell. Homologous recombination deficiency (HRD) mostly affects the *BRCA1* and/or *BRCA2* genes, and in 6–27%, they affect the *RAD 51D*, *ATM*, *NBN*, and *PALB2* genes. In addition to *BRCA1/2* mutations, mutations in other genes such as *MLH1*, *MSH2*, *MSH6*, *EPCAM*, and *PMS2* are also associated with the risk of ovarian cancer. They are responsible for hereditary non-polyposis colorectal cancer (HNPCC). Other mutations responsible for the development of this cancer are germline mutations in the *BRIP1*, *RAD51C*, or *RAD51D* genes [47,48].

In the case of patients with ovarian cancer, it is recommended to start molecular diagnostics with *BRCA1/2* gene sequencing. This is related to the growing importance of PARP inhibitors in cancer therapy, and the presence of *BRCA1* or *BRCA2* mutations is a good predictive marker that indicates the probable high effectiveness of PARP inhibitors in therapy. Modern treatment with a PARP inhibitor for a group of patients with hereditary *BRCA1/2* has been reimbursed in Poland for several years. However, the remaining 75–80% of patients (without the presence of mutations in the *BRCA1/2* genes) could not count on a similar standard of treatment so far. As of January 2022, niraparib has been included on the list of reimbursed drugs (in Poland) for the indication of ovarian cancer without mutations in the *BRCA1/2* genes. In the group of patients using niraparib, a significantly longer time to progression and a longer time to subsequent treatment was demonstrated than in the placebo group. Maintenance therapy with niraparib currently seems to be a very good therapeutic option [49].

Advanced ovarian cancer returned in 80% of patients before the use of PARP inhibitors. There is no data yet on how this group of drugs will change the landscape in the case of ovarian cancer without *BRCA1/2* mutations, but based on the results of clinical trials, it is assumed that patients will live much longer. Studies show that the use of PARP inhibitors prolongs the progression-free time of the disease. In addition, as a result of the identification of *BRCA1* or *BRCA2* mutations, the verification of the nature of the detected change is required (germline mutation–hereditary or somatic–non-hereditary). This verification is carried out by analyzing its presence in DNA isolated from cells outside the tumor. In addition to patients with ovarian cancer, genetic counseling should be provided to women with ovarian cancer in whom no mutation in tDNA has been identified and women in whom molecular testing for tDNA has not been performed. Each patient with ovarian cancer referred to genetic clinics undergoes a pedigree–clinical analysis and differential diagnosis, including hereditary non-polyposis colorectal cancer (HNPCC, Lynch syndrome). In patients who have not been sequenced for *BRCA1* and *BRCA2* per tDNA, a genetic test for constitutional genome DNA for the five founding mutations of the *BRCA1* gene is recommended. If patients have a family history, the test is then expanded to include *BRCA1/2* gene sequencing. As a result of the above activities, individual preventive and therapeutic recommendations are given. If patients with ovarian cancer have a hereditary burden of cancer and do not have a *BRCA1/2* mutation, they may undergo broad-panel, commercially available, next-generation genetic sequencing (NGS) testing. These tests allow for the sequencing of many genes associated with different syndromes of a hereditary predisposition to cancer within a single test. However, all genetic tests have limitations. Therefore, if no mutation is found, it is not a basis for ruling out a suspected hereditary cancer predisposition syndrome. Therefore, the final recommendations should be formed as a result of a full comprehensive analysis, which takes into account the results of molecular tests and pedigree–clinical assessment. If a patient is diagnosed with a terminal (hereditary) mutation, she should be made aware of the risk of its occurrence in the family. Genetic counseling should be provided not only to the patient, but also to selected relatives [50].

## 10. The Role of Estrogen Receptors in Ovarian Cancer Oncogenesis

The ovaries are the central reproductive organs of women. They produce hormones such as testosterone, progesterone, and estrogen. Estrogens include estrone (E1), 17β-estradiol (E2), and estriol (E3), of which E2 is the most dominant estrogen [51]. Estrogen cell signaling is mediated by the ERs. ERs are a family of transcription factors that control the biological function of estrogens by regulating gene transcription using estrogen response elements (EREs) [52]. Among estrogen receptors, we can distinguish both classical receptors (ERα and ERβ) and non-classical receptors (estrogen receptor coupleds to the G 1 protein (GPER1)) [53,54,55]. Estrogens may modulate the biology of cancer cells by affecting processes like cell proliferation, invasion, apoptosis, the cell cycle, and inflammation [56].

**Figure 7:**
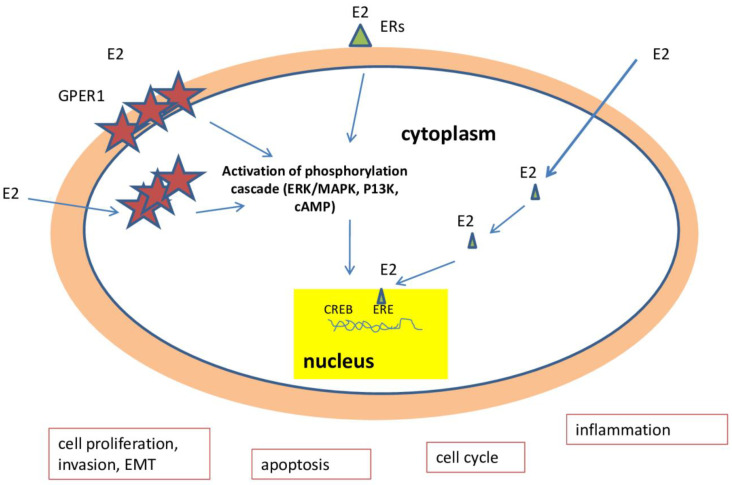
A schematic representation of the main mechanisms of estrogen-induced changes in the biology of ovarian cancer cells. E2—estradiol. ERs—estrogen receptors. EMT—epithelial to mesenchymal transition. GPER1—G-protein-coupled estrogen receptor 1. ERE—estrogen response element. CREB—cAMP-response-element-binding protein.

The main mechanisms of changes in the biology of estrogen-induced ovarian cancer cells are presented by Figure 7.

Although ovarian cancer is not one of the most common cancers affecting women, it is one of the deadliest, which is caused by too-late diagnoses. Therefore, research is being conducted to learn about new predictive markers for this disease. Studies pay attention to estrogen receptors.

The ratio between estrogen receptors (ERα and ERβ) plays a significant role in the development of ovarian cancer. The alpha receptor is encoded by the *ESR1* gene located on chromosome 6q25.1, and the beta receptor is encoded by the *ESR2* gene on chromosome 14q23. They belong to the family of nuclear receptors [57,58].

Both receptors can activate or inhibit the transcription process of many genes. They can also act antagonistically to each other after binding the same ligand [59].

The initiation of the transformation process is the result of the combination of estrogens with the receptor protein, as a result of which, the conformation changes and the part of the receptor that binds to DNA is exposed. Both alpha and beta estrogen receptors belong to ligand-activated transcription factors. Because the two receptors exhibit different and distinct function, tissue distribution, and bind different cofactors, the cell’s response to estrogen is diverse [60].

The ER-α estrogen receptor is a protein transcription factor activated by the ligands 17β-estradiol, estrone, and estriol [61]. In the body, 17β-estradiol and estrone are produced by the granulosa cells of the ovarian follicle under physiological conditions. In postmenopausal women diagnosed with epithelial ovarian cancer, elevated levels of these hormones in the peripheral circulation are detected. It has been shown that increased levels of estrogen in the blood are the result of their production by ovarian cancer cells [62,63,64].

In ovarian epithelial cells and ovarian tumors, the aromatase gene is expressed. To sum up, estrogens create a hormonal environment optimal for the development of cancer. They actively participate in the process of regulating the multiplication of cancer cells [63].

In normal cells of both the ovarian epithelium and in the cells of epithelial ovarian cancer, the presence of estrogen receptors was found. In 60% of ovarian cancer cases, significantly elevated levels of ERα receptors were found [62,63,65].

ER-α is present in the largest amount in the cell nucleus and less in the cytoplasm. ER-α is also found in the cell membrane of some cells [66].

Estrogens work through two mechanisms. The first is by directly binding to DNA through the estrogen responsive element (ERE) and also by an indirect mechanism via the transcription factor AP1. The specific site in DNA that interacts with the estrogen receptor is the ERE. The second mechanism is the action of the activated estrogen receptor through the expression enhancer of the AP1 type [67,68].

The expression of estrogen receptors alpha and beta in ovarian cancer cells is dependent on histopathological type. The occurrence of estrogen receptors was analyzed in various histopathological types of ovarian cancer [69].

The ER-alpha receptor was found in 97% of serous neoplasms with adenocarcinoma, in 100% of endometrioid neoplasms, and in 70% of mucinous neoplasms [62]. There was no expression of the ER-alpha receptor in the clear cell adenocarcinoma type. The ER-beta receptor was present in all the histopathological types of ovarian cancer: serous carcinoma 41%, mucinous carcinoma 30%, endometrioid carcinoma 75%, and clear cell carcinoma 39% [62].

The different expression of ER-α and -β receptors has been demonstrated between normal ovarian epithelium, primary, and metastatic ovarian cancer tumors [70,71]. In normal epithelium, ER-α and -β receptors were expressed, and in primary tumors, the reduced expression of ER-α receptors was observed, while in metastatic ovarian cancer tumors, the lack of the expression of the ER-β receptor was found [64,65].

Studies on ovarian cancer cell lines lacking the ER-α receptor (PEO14) and containing the ER-α receptor (BG1) have shown that ER-α can affect the progesterone receptor, while ER-β cannot [72,73].

The opposite effects of ER-α and –β on the activity of cyclin D1, a cell cycle protein, were found [74]. ER-β decreases the expression of the cyclin D1 gene, while ER-α increases its levels [75].

The increased expression of ER-β inhibits the proliferation and reduces the motility of ovarian cancer cells. ER-α does not affect these processes. ER-β is an important regulator of the proliferation and motility of ovarian cancer cells and has a pro-apoptotic effect. The loss of ER-β receptor expression may be a significant event leading to the development of ovarian cancer [63].

The following results were obtained in meta-analysis studies on the frequency of the expression of sex hormone receptors in serous ovarian carcinoma [76]. Low-grade serous ovarian carcinoma expressed estrogen receptors in 81% of cases and progestogen receptors in 54% of cases. For high-grade serous carcinoma, the percentages were 62% and 31%, respectively.

Low-grade serous carcinoma expresses sex hormone receptors in a high percentage. This type of tumor is more indolent and less sensitive to chemotherapy. Therefore, the use of hormone therapy could be a therapeutic option for this tumor. In the case of serous carcinoma, when a high degree of malignancy is observed, there is dynamic growth and greater sensitivity to chemotherapy. It is suggested that in such a large proportion of patients, additional hormone therapy could be used.

The primary clinical significance of estrogen receptor polymorphisms α is related to its use as a predictive factor, enabling therapeutic decisions. The research work currently being carried out focuses on the designation of several basic polymorphisms: PvuII, XbaI, and others that are much less frequently determined (rs746432, rs2077647, and rs532010). They include the assessment of the importance of these polymorphisms in endometrial diseases [77]; osteoporosis [78,79]; and prostate [80], breast [81,82,83], and ovarian cancer [84].

## 11. Single Nucleotide Polymorphisms

Polymorphism is when a genetic change (a given allele of a specific gene) occurs in a population with a frequency of more than 1%. We distinguish single nucleotide polymorphisms and mini- and microsatellite sequence polymorphisms [85,86,87].

**Figure 8:**
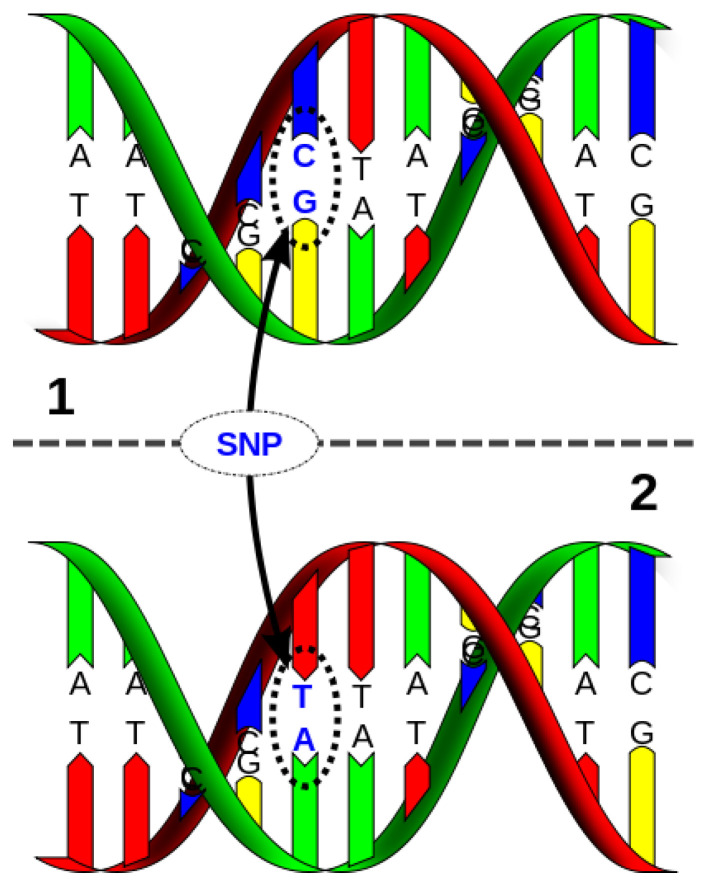
Single nucleotide polymorphism.

SNPs are the phenomenon of DNA sequence variation. It is the replacement of one nucleotide (G, C, T, or A) between individuals of a given species or another corresponding chromosome of a given individual [88,89,90]. A schematic representation of an SNP is presented in Figure 8.

In the process of neoplastic transformation, in addition to mutations in proto-oncogenes and tumor suppressor genes, genetic polymorphisms that determine a change in the amino acid sequence of proteins or affect the binding site of transcription factors may also be important [91].

These polymorphisms, including single nucleotide polymorphisms, can affect both the change in the risk of cancer and the effectiveness of therapy, playing an important role in the transport, metabolism, tissue distribution, and elimination of the drug [92,93].

Polymorphisms in genes encoding hormone receptors were correlated with changes in reproductive factors and an increased risk of ovarian cancer in different populations. Among hormone receptors, the estrogen receptor has been extensively studied for different ethnic groups. Estrogens are a group of compounds of particular importance for reproductive processes. The effects of estrogen are stimulated by the estrogen receptor, a dimeric nuclear protein that binds to DNA and controls gene expression. ER expression occurs in specific tissues: ovary, uterus, and breast [94,95].

Estrogen’s increased affinity for binding to its receptor is a key mechanism in controlling biological effects [96,97].

**Figure 9:**
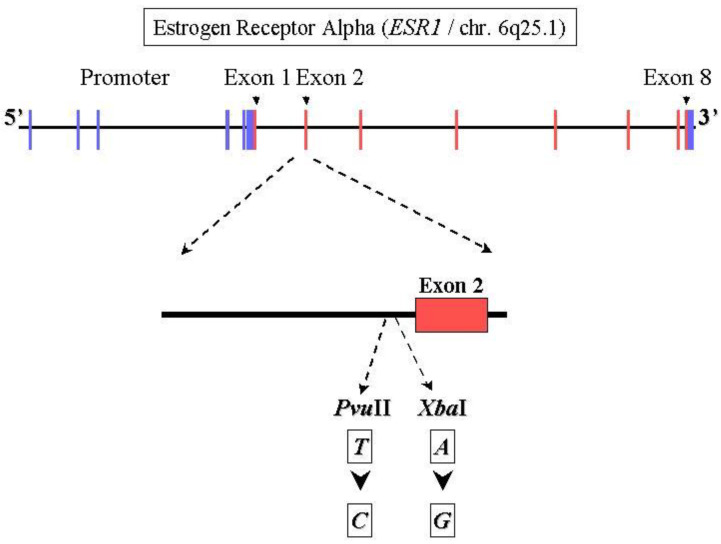
The organization of the estrogen receptor α gene with the position of the Xbal and PvuII polymorphic variants.

In normal cells of both the ovarian epithelium and in the cells of epithelial ovarian cancer, the presence of estrogen receptors was found. In 60% of cancer cases, significantly elevated levels of ERα receptors were detected [98,99,100,101]. The ERα receptor is encoded by the *ESR1* gene. *ESR1* is located on chromosome 6q25.1 and consists of eight exons. The first intron and the gene promoter contain regulatory sequences for other introns. Several single nucleotide polymorphisms and a variable number of tandem repeat polymorphisms (VNTRs) have been identified in *ESR1*. The two polymorphisms that affect ER activity, the most commonly studied in the *ESR1* gene, are SNPs, rs2234693 (PvuII) and rs9340799 (XbaI), which remain in a coupling disequilibrium with the VNTR polymorphism in the promoter region [102] (Figure 9).

The polymorphic variant of the *ESR1* rs2234693 (PvuII) gene is located above exon 2, in the first base pair of an intron of 397 bps length, in which cytosine is replaced by thymine, and this change is identified by PvuII endonuclease [103,104]. The rs9340799 polymorphism (XbaI), located 50 bp from the polymorphic site PvuII, [105] is a variant in which guanine is replaced by adenine. In the case of the *ESR1*PvuII gene polymorphism, it has been shown that the C allele forms part of the functional binding site of the transcription factor B-myb and acts as an intragenic enhancer [106]. Estrogen is thought to be involved in the regulation of myb transcription, [107]. The presence of a PvuII site corresponding to the T allele may lead to the reduced expression of *ESR1*, and thus the effects of estrogen through the indirect action of *ESR1* may be reduced, resulting in relative estrogen deficiency. The XbaI polymorphism may also have a functional significance that remains unclear. Polymorphisms in intron regions have been reported to modify splicing in mRNA transcripts, leading to significant changes in gene function [108]. However, differences in the incidence of these polymorphisms have been described, and depending on the population studied, the T or C allele has been identified as a protective or risk factor for breast cancer [109,110].

The polymorphisms rs9340799 and rs2234693 are in a strong coupling imbalance with each other. At this time, there is no biological evidence for the functionality of these SNPs, although recent findings support the hypothesis that the above polymorphisms affect estrogen activity, as a result of the regulation of the transcription of the *ESR1* gene by modulating transcription factor binding. Association studies remain controversial for a variety of reasons: one is the difficulty of determining the disease-related allele, and the other is the genetic variation found in each ethnic population and the specificity of each study. At present, the research work focuses on the analysis of several basic polymorphisms: PvuII, XbaI, and others much less frequently determined (rs746432, rs2077647 and rs532010). They include the assessment of the significance of these polymorphisms in the case of hormone-dependent cancers, such as endometrial cancer [111], breast cancer [112,113,114], or ovarian cancer [84,115]. *ESR1* polymorphisms have also been reported to be involved in the response to ovarian hyperstimulation in assisted reproductive studies [116]. The presence of the rs2234693 polymorphism of the *ESR1* gene may be one of the risk factors for the development of ovarian cancer in the Indian population [84]. It was shown that patients with ovarian cancer were carriers of the TT genotype in 50%, TC heterozygotes in 33.75%, and carriers of CC homozygotes in 16.25%. In the case of the control group, TT homozygotes accounted for 79%, the TC genotype accounted for 12%, and the CC homozygotes accounted for 9%. The patients with ovarian cancer had a significant increase in the frequency of allele C compared to the controls. In the case of the rs9340799 polymorphism, the presence of the AG genotype (70%) was found to prevail in the patients with ovarian cancer [117]. Most of the studied patients with ovarian cancer were carriers of the G allele, while the patients of the control group were carriers of the A allele. Researchers suggest that the AG and GG genotypes may be a risk factor for ovarian cancer.

Genetic studies of *ESR1* polymorphisms in cancer are still ongoing. Epidemiological studies were conducted to assess the association of the *ESR1* PvuII polymorphism with the risk of cancer. The results showed no significant association between the overall cancer risk and PvuII polymorphism in homozygous (TT vs. CC) and heterozygous (TT vs. CT) models. A statistically significant relationship was observed only for the PvuII polymorphism in the T vs. C allele model. The analysis of stratification by the tumor type suggested that the T genotype significantly reduced the risk of prostate cancer, the risk of leiomyoma, and the risk of hepatocellular carcinoma. The results suggest that the *ESR1*PvuII polymorphism (rs2234693 T>C) may have only a small effect on cancer susceptibility [118]. Large-scale epidemiological studies are warranted in the future to verify these results. In the work of Kutilin et al., genes regulating apoptosis, DNA repair, cell proliferation, estrogen metabolism and regulation in cancer, and normal serous ovarian adenocarcinoma of high and low grades were analyzed [119]. Using real-time qPCR, the relative copy number of 34 genes (*BAX*, *BCL2*, *TP53*, *MDM2*, *CASP9*, *CASP3*, *CASP7*, *CASP8*, *PRKCI*, *SOX2*, *OCT4*, *PIK3*, *PTEN*, *C-MYC*, *SOX18*, *AKT1*, *NOTCH1*, *BRCA1*, *BRCA2*, *EXO1*, *SCNN1A*, *KRAS*, *EGFR*, *BRAF*, *CYP1A1*, *CYP1A2*, *CYP1B1*, *CYP19A*, *ESR1*, *ESR2*, *GPER*, *STS*, *SULT1A*, and *SULT1E1*) was determined in normal and neoplastic ovarian cells collected by a non-contact laser microscope from FFPE paraffin blocks from 200 patients. The most typical molecular markers of serous ovarian adenocarcinoma cells were identified: the copy number of the *PIK3CA*, *BCL2*, *BAX*, *CASP3*, and *CASP8* genes. Based on the differences in the gene copy number variation, two molecular subtypes of serous adenocarcinoma were identified, corresponding to two histological subtypes: high-grade (*MDM2*, *SOX2*, *ESR1*, *CYP1B1*, *SULT1E1*, *TP53*, and *BRCA2*) and low-grade (*PIK3CA*, *PTEN*, *BCL2*, *BAX*, and *CASP3*). Each of these subtypes was also characterized by molecular heterogeneity and can be divided into several subgroups: three subgroups for high-grade and four subgroups for low-grade serum adenocarcinoma. These results expand our understanding of the molecular mechanisms of carcinogenesis in ovarian tissue and confirm the molecular differences between the two histological subtypes of serous adenocarcinoma, which probably underlie their different clinical courses. There are reports indicating that mutations in the *ESR1* gene may affect endocrine therapy, often translating into its resistance [119]. It is known that gynecological cancers, such as ovarian cancer and low-grade endometrial cancer, are often characterized by the expression of the estrogen receptor ERα, encoded by *ESR1*. These are hormone-sensitive cancers. In the case of these cancers, the use of endocrine therapy in the advanced and recurrent stages of the disease is common. Studies indicate that mutations in the *ESR1* gene may be a mechanism of resistance to aromatase inhibitor treatment in gynecological cancers. It was shown that the most common mutations were L536, Y537, and D538, which correlated with the higher expression of *ESR1* mRNA [120].

Patients with endometriosis are at risk of developing certain types of histological ovarian cancer. The risk of developing ovarian cancer is four times higher in women with endometriosis compared to women who have not been diagnosed with the disease. In the case of its severe subtypes, the risk increases up to nearly 10-fold [121]. In the latest study, scientists from the United States analyzed data on almost 500,000 women in the state of Utah, aged 18 to 55. For the first time, the incidence rates of different types of endometriosis and subtypes of ovarian cancer were looked at. The risk of type I ovarian cancer was “particularly high” (about 7.5 times higher in women with endometriosis), and the risk of type II ovarian cancer—which can be more aggressive—was about 2.7 times more likely. It has been suggested that such a population should benefit from ovarian cancer risk counseling and prevention and may be an important group for targeted screening and prevention [122]. At present, the study of variants in the PvuII and XbaIlocis in endometriosis is the subject of scientific debate.

The data reported in the literature indicate that endometriosis is affected by an increase in estradiol levels and ERβ expression, polymorphic genotypes and alleles of the ERβ rs4986938 G/A gene, and the frequency of alleles of the ERα rs9340799 A/G gene [123].

In a meta-analysis study involving 4975 patients (2222 patients with ovarian cancer, 2753 controls), *ESR1*Pvull/Xbal polymorphisms were analyzed. The total combined results showed no significant correlations between ESR1 Pvull/Xbal polymorphisms and the development of endometriosis. In the subgroup analysis, PvuII was associated with endometriosis only in stages I–III and only in the recessive model. Xbal was associated with endometriosis only with the PCR-RFLP genotyping method and also only in the recessive model. The meta-analysis showed that PvuII or Xbal polymorphisms were not associated with susceptibility to endometriosis except for a small association of endometriosis in stages I–III and PCR-RFLP in a recessive model. The results suggest that further, well-designed, large-scale studies are needed to shed light on the problem of ovarian cancer formation due to endometriosis in correlation with *ESR1* polymorphisms [124].

Ovarian cancer, due to the lack of specific symptoms at an early stage of the disease, is very often diagnosed at a very advanced stage, with a poor prognosis. Therefore, research has been carried out for a long time to obtain an ideal marker of this cancer. Despite significant achievements and the use of tests, such as the previously described ROMA or Ova1 (based on various biomarkers, including CA-125, it was developed to estimate the nature of the tumor to determine the probability of malignancy even before surgical treatment and histopathological examination), effective methods for the early diagnosis of ovarian cancer are still being sought. Despite a relatively strong genetic predisposition to ovarian cancer, only a few variants associated with this cancer have been discovered so far. It seems that this may be related to the low penetration of genes responsible for the development of cancer. Single nucleotide polymorphisms may constitute a group of new risk factors for cancer development, and their analysis may be used to qualify patients to the group of patients with a high risk of developing the disease. In addition, they can be extremely useful in assessing the individual risk of developing the disease in people without symptoms of cancer. The results of the research may be used in the future to improve earlier cancer diagnosis and, as a result, to extend the survival time of patients with these cancers.

## 12. Summary

According to statistical data from recent years, the incidence of ovarian cancer in the world is constantly increasing. Very diverse incidence is observed across continents, geographical regions, races, and ethnic groups. These cancers most often affect women after menopause and are usually diagnosed (70%) at late stages. Despite improvements in surgical techniques and the introduction of new chemotherapy methods, the five-year survival rate for ovarian cancer patients has remained essentially unchanged and now stands at around 30–40%. Epidemiological analysis distinguishes a number of factors related to the incidence of ovarian cancer. The degree of advancement is the most important prognostic factor. In order to assess the clinical stage of malignant ovarian cancers, the International Association of Gynecology and Obstetrics (FIGO) has developed an appropriate classification of these cancers. Ovarian tumors are characterized by a large variety of morphological and biological features. Based on the established clinical stage and maturity, the optimal method of surgical treatment and chemotherapy is selected in order to achieve the highest possible cytoreduction of the tumor. About 5–10% of ovarian cancer cases are hereditary. Two ovarian cancer susceptibility genes have been located: *BRCA1* and *BRCA2*, whose mutations are inherited in an autosomal dominant manner. It should be added that studies of the above-mentioned genes do not explain all familial aggregations of ovarian cancer cases. An alternative model is to assume a combination of numerous, frequent, low-risk genetic variants, for example different polymorphic alleles. In 60% of ovarian cancer cases, significantly elevated levels of ERα receptors are detected. The ERα receptor is encoded by the *ESR1* gene, so its polymorphisms can be studied as potential molecular markers for ovarian cancer. The studied single nucleotide polymorphisms may constitute a group of new risk factors for cancer development, and their analysis can be used to qualify patients to the group of patients with a high risk of developing the disease. In addition, they can be extremely useful in assessing an individual’s risk of developing the disease in people without symptoms of cancer. The results of the research in the future may be used to improve the earlier diagnosis of cancer and, as a result, extend the survival time of patients with these cancers.

## Figures and Tables

**Table 1:** Interpretation of ROMA test results.

Value of ROMA Test	Risk of Ovarian Cancer
premenopausal women	
<11.4%	Low
>11.4%	High
postmenopausal women	
<29.9%	Low
>29.9%	High

**Table 2:** Stages of ovarian cancer, according to FIGO [33].

Degree of Advancement	Description
I	The cancer is limited to the ovaries or fallopian tubes.
IA	A tumor confined to one ovary or fallopian tube (intact continuity of the tumor capsule), no lesions on the surface of the ovary or the fallopian tube, an absence of tumor cells from fluid or peritoneal lavages.
IB	Tumors limited to 2 ovaries or fallopian tubes (intact continuity of the tumor capsule), no lesions on the surface of the ovary or the fallopian tube, no tumor cells in the fluid or peritoneal lavages.
IC	Tumors confined to 1 or 2 ovaries or fallopian tubes with the following:
IC1	An intraoperative capsular injury;
IC2	Impaired capsule continuity prior to surgery or the presence of a tumor on the surface of the ovary or fallopian tube;
IC3	The presence of cancer cells in the fluid or peritoneal lavage.
II	Cancer limited to the ovaries or fallopian tubes with the involvement of the pelvic structure (below the plane of pelvic entry) or primary peritoneal cancer.
IIA	Involvement and/or implantation on the surface of the uterus and/or fallopian tube and/or ovaries.
IIB	The involvement of other pelvic structures.
III	Neoplasm involving 1 or 2 ovaries/fallopian tubes or primary peritoneal cancer with metastases to the peritoneum outside the pelvis and/or metastases to retroperitoneal lymph nodes.
IIIA1	Neoplastic metastases are present only in the retroperitoneal lymph nodes (histopathologically confirmed).
IIIA1(i)	Metastases to the retroperitoneal lymph nodes in the largest dimension ≤ 10 mm.
IIIA1(ii)	Metastases to the retroperitoneal lymph nodes in the largest dimension > 10 mm.
IIIA2	Microscopic peritoneal metastases outside the lesser pelvis (above the plane of pelvic entry), with or without retroperitoneal lymph node metastases.
IIIB	Macroscopic peritoneal metastases outside the lesser pelvis with a diameter of ≤2 cm in the largest dimension, with or without retroperitoneal lymph node metastases.
IIIC	Macroscopic peritoneal metastases outside the pelvis with a diameter of >2 cm in the largest dimension, with or without retroperitoneal lymph node metastases (including the involvement of the liver capsule and the spleen by neoplasm, without the infiltration of the parenchyma of the organ).
IV	The presence of distant metastases (including peritoneal metastases).
IVA	Pleural effusion with cytologically confirmed neoplasm.
IVB	Interstitial liver metastases and organ metastases outside the abdomen (including inguinal lymph nodes and lymph nodes outside the abdominal cavity).

**Table 3:** Hormonal treatment options for ovarian cancer.

HTH	Mechanism of Action	Options
Gonadotropin-releasing hormone receptor analogs	Competitively binds GnRH-R and reduces the secretion of follicle-stimulating hormone and luteinizing hormone	GnRH I agonists Triptorelin Goserelin Histrelin Leuprolide acetate GnRH II antagonists Cetrorelix Degarelix acetate
Estrogen	Estrogen receptor blockade	Antiestrogens: Tamoxifen Toremifene
	Estrogen synthesis suppression	Aromatase inhibitors: Anastrozole Exemestane Letrozole
	Estrogen receptor downregulation	ER antagonist: Fulvestrant
	Hormonal ablation	Surgery Radiation (infrequently used)
Androgen	Androgen receptor blockade	Anti-androgens: Flutamide Bicalutamide Enzalutamide
Progesterone	Progesterone receptor blockade	PR antagonists: Mifepristone Medroxyprogesterone Megestrol acetate
	Increasing progesterone levels	Oral contraceptive pills Pregnancy Breastfeeding

**Table 4:** Drugs and dosages used in the treatment of ovarian cancer.

Medicines	Drug Doses	Indicate
Olaparib	200–800 mg per day	Indicated for the maintenance therapy of recurrent serous ovarian cancer. The presence of BRCA mutations should be confirmed prior to use.
Lynparz	300 mg twice daily	Indicated as monotherapy for The maintenance treatment of adult patients with advanced (FIGO grade III and IV) low-differentiated epithelial ovarian cancer, fallopian tube cancer, or primary peritoneal cancer with BRCA1/2 mutations (hereditary and/or somatic) who have a response (complete or partial) following the completion of platinum-based, first-line chemotherapy. The maintenance treatment of adult patients with high-grade, platinum-sensitive, recurrent ovarian cancer, fallopian tube cancer, or primary peritoneal cancer who have responded (complete or partial) to platinum-based chemotherapy.
Tamoxifen (Selective Estrogen Receptor Modulators)	20–40 mg per day	It is one of the types of hormonal drugs, i.e., the so-called antiestrogens, and is used in the treatment of hormone-dependent cancers. These include breast cancer (breast cancer), ovarian cancer, and prostate cancer.
Letrozole (Aromatase Inhibitor)	2.5 mg daily	Letrozole is a drug from the group of aromatase inhibitors. It works by blocking the aromatase enzyme, which leads to a decrease in the production of estrogen in the body. Estrogen is a hormone that can accelerate the growth of certain cancers. By lowering estrogen levels, letrozole can slow or stop the growth of these cancer cells.
Cetrorelix (Antagonist of GnRH)	0.25 mg per day	Its main function is to inhibit the secretion of luteinizing hormone from the anterior pituitary gland, thereby preventing ovulation and inhibiting the production of sex steroids.
